# Retraction Note: Children’s physical activity level and sedentary behaviour in Norwegian early childhood education and care: effects of a staff-led cluster-randomised controlled trial

**DOI:** 10.1186/s12889-024-18629-0

**Published:** 2024-04-19

**Authors:** Eivind Andersen, Steinar Øvreås, Kari Anne Jørgensen, Janne Borch-Jenssen, Thomas Moser

**Affiliations:** 1 Faculty of Humanities, Sports and Educational Science, University of South-Eastern Norway, Horten, Norway; 2 Sandefjord Municipality, Sandefjord, Norway

**Retraction Note** **:** **BMC Public Health 20, 1651 (2020)**

**https://doi.org/10.1186/s12889-020-09725-y**

The Editors have retracted this article. After publication, concerns were raised about the statistical significance of the findings. Post-publication review found additional issues such as excluding individuals with missing observations, testing repeating physical activity (Fig. 3) instead of repeated measure analysis, and lack of clarity about how the accelerometer data was summarised and assessed as the results were not easily reproducible. Therefore, the Editors have lost confidence in the findings of this study. The authors did not state explicitly whether they agree to this retraction.

